# Attitudes of Israeli Rheumatologists to the Use of Medical Cannabis as Therapy for Rheumatic Disorders

**DOI:** 10.5041/RMMJ.10239

**Published:** 2016-04-19

**Authors:** Jacob N. Ablin, Ori Elkayam, Mary-Ann Fitzcharles

**Affiliations:** 1 Institute of Rheumatology, Tel Aviv Sourasky Medical Center, Tel Aviv, Israel; 2 Sackler School of Medicine, Tel Aviv University, Tel Aviv, Israel; 3 McGill University Health Centre, Montreal, Quebec, Canada

**Keywords:** Medical cannabis, opinion survey, rheumatology

## Abstract

**Background:**

While medical cannabis has been used for thousands of years in the treatment of pain and other symptoms, evidence-based use is limited and practitioners face multiple areas of uncertainty regarding the rational use of these compounds. Nonetheless, an increasing public interest and advocacy in favor of medical cannabis is causing the issue to be encountered ever more frequently by physicians in different fields of medicine and particularly in rheumatology. In view of this situation, we have surveyed the attitudes of Israeli rheumatologists to the use of medical cannabis.

**Objectives:**

As rheumatologists are specialized in caring for patients presenting with musculoskeletal complaints, the confidence of rheumatologists’ knowledge of cannabinoids was surveyed.

**Methods:**

All members of the Israeli Society of Rheumatology were surveyed by e-mail for their confidence and knowledge of cannabinoids and their perceived competence to prescribe herbal cannabis.

**Results:**

A total of 23 out of 119 (19.3%) Israeli rheumatologists approached returned the questionnaire. Three-quarters of responders were not confident about their knowledge of cannabinoid molecules or ability to write a prescription for herbal cannabis, and 78% were not confident to write a prescription for herbal cannabis; 74% of responders held the opinion that there was some role for cannabinoids in the management of rheumatic disease.

**Conclusion:**

Israeli rheumatologists lack confidence in their knowledge of cannabinoids in general, yet are open to the possibility of introducing this treatment. Additional data and guidance are necessary in order to allow rational utilization of cannabinoids for management of rheumatic pain.

## BACKGROUND

Cannabis has been used for millennia in the treatment of pain, as well as for other indications.^1^ Well up into the twentieth century cannabis was considered an integral part of the medical armamentarium and was the subject of extensive research. However, this course changed sharply during the 1930s, with the de facto criminalization of cannabis in most parts of the Western world.^2^,^3^ Thus, generations of medical professionals have been educated in an environment in which cannabis is considered to be a purely illicit drug, with no legitimate medical use (as defined in the USA by the term “schedule 1”). Cannabis indeed continues to be widely used illegally for recreational purposes all around the world.^3^

Despite this history, recent decades have witnessed a significant change in the attitude of the medical community toward cannabis and cannabinoids. Medical cannabis was originally introduced into practice in various countries mainly as treatment for terminally ill patients suffering from cancer or AIDS. Subsequently, however, indications for the use of medical cannabis have broadened significantly in some countries, while other countries retain an extremely restrictive attitude. Thus, cannabis is being increasingly used for the treatment of chronic non-malignant pain such as pain associated with chronic arthritis or fibromyalgia.^4^

While this shift is given significant backing by public opinion in many places, the medical use of cannabis remains in many ways different from most classical modalities of treatment. Evidence-based medicine is all but non-existent in this context. Randomized controlled trials are few and difficult to perform. Furthermore the use of extracts made out of different strains of *Cannabis sativa* implies that different patients eventually are receiving completely different combinations of the various (and numerous) active ingredients, e.g. tetrahydrocannabinol and cannabidiol.^3^ In addition, different modes of administration, ranging from smoking to eating cookies, lead to different clinical results. The idea of prescribing “smoking a joint” appears to be particularly unappealing for many physicians, who are trained in explaining the multiple dangers of smoking.

On the other hand, treating chronic pain remains an unmet need, and many patients continue to suffer from uncontrollable pain to an extent incompatible with productive life and function.^5^,^6^ This situation persists despite advances made in understanding the mechanisms of pain centralization and its role in rheumatic disease,^7^–^9^ and despite the introduction of a number of new classes of medications for chronic pain. Thus, the need to find effective tools for fighting chronic pain (with minimum side effects) is a high priority for patients and health care providers alike.

Israel, together with a number of other countries, such as Canada, has been a leader in the introduction of medical cannabis for non-malignant indications, as well as becoming a significant producer of medical cannabis.^10^ This process has been accompanied by an increasingly outspoken public campaign, voicing a stance for more rapid introduction and facilitation of the use of medical cannabis, and/or for the decriminalization and eventual legalization of recreational cannabis. This has naturally led to an increase in the number of patients requesting medical cannabis.

Such significant changes in public attitude and in the legal milieu are often met with confusion and concern within the medical community, which is called upon rapidly to adjust to the new circumstances while simultaneously struggling to preserve medical integrity and professionalism. Since many patients applying for cannabis do so on the grounds of “chronic arthritis,” rheumatologists find themselves at the forefront of this shifting reality and are challenged by it. Rheumatologists in other countries such as Canada have previously been surveyed and reported a lack of confidence in their knowledge of cannabinoid molecules and ability to advise patients on use of either herbal or pharmaceutical cannabinoids.^11^ Thus, in the current study we have surveyed the attitudes of Israeli rheumatologists toward the use of medical cannabis in order to gauge the situation in the Israeli reality and in order to focus attention on the implications of these attitudes.

## MATERIALS AND METHODS

The study was designed as a follow-up to the above-mentioned Canadian survey and aimed, among other goals, to achieve a cross-cultural comparison between the situation in Israel and Canada on this issue. Thus, the methodology, including the survey questionnaire, was adapted from the Canadian study with participation of one of the authors (M.-A.F.).

In June 2013 the entire Israeli Society of Rheumatology membership, comprising 119 rheumatologists, was invited via e-mail to participate in a survey examining current knowledge and perceptions regarding cannabinoid use in rheumatology patients. The survey was distributed through the secretariat of the Israeli Society of Rheumatology, and Israeli rheumatologists were encouraged to participate in the survey at the annual meeting of the Israeli Society. Data were collected once the survey was closed.

As previously described,^11^ the 19-question survey comprised three sections. The first obtained demographic information, years and location of practice, type of practice (academic, community, or private), and age and gender. The second addressed respondents’ current perception of knowledge of cannabinoids including phyto-, syntheto-, and endocannabinoids, as well as respondents’ belief whether there is a role for cannabinoids in general in rheumatology practice. The third addressed physicians’ response regarding interactions with patients requesting cannabinoids or those in whom other pain treatments had failed. The entire questionnaire was translated into Hebrew by one of the authors (J.N.A.), who is fluent in Hebrew and English. Perception of knowledge of cannabinoids was assessed according to confidence (very confident; confident; somewhat confident; not confident). Respondents reported whether they had previously recommended trials of cannabinoids to treat rheumatic pain, and whether they would consider a cannabinoid trial in the future.

Specific questions regarding the use of medical cannabis were as follows:

- Do you believe there is a role for use of medical cannabis in treating rheumatic diseases?
- How would you react if a patient asked about use of medical cannabis?
- Have you ever recommended a trial of medical cannabis or would you recommend such a trial in the future?
- In the event of recommending medical cannabis, what is your level of confidence in writing a prescription regarding dosage, frequency of use, and method of administration?
- If a prescription was written, what would be the starting dose, maximum dose, dose frequency, and method of administration?

Narrative responses were obtained covering the following areas: variables needing consideration in patients requesting medical cannabis; advice given regarding precautions when using medical cannabis; and patient characteristics that would be a barrier to a prescription of medical cannabis.

## RESULTS

### Physician Demographics

The survey was sent to all 119 members of the Israeli Society of Rheumatology, who are either specialists in rheumatology or training in rheumatology. A total of 23 physicians returned the questionnaire for a response rate of 19.3%. Both specialists in rheumatology and rheumatology residents were included.

Demographic information for the respondents is shown in Table 1. As presented in the table, the majority of respondents were over the age of 35; 78% were male. Most had been in practice for over 10 years, and 83% reported that their practice was academic or a combination of academic and private practice.

**Table 1:** Demographic Characteristics of Israeli Rheumatologists Completing the Study Survey.

	Characteristics	*n* (%)*
**Gender**	Male	18 (78)
	Female	5 (22)

**Age**	35 or younger	1 (4)
	36–49	13 (57)
	50 and over	9 (39)

**Years in Practice**†	<10	1 (4)
	10–24	11 (48)
	25 or over	9 (39)

**Practice Type**	Academic	4 (17)
	Academic/private	4 (17)
	Community hospital/private	15 (65)
	Private	0 (0)

* Total respondents: *n*=23, except “Years in practice”;

† Respondents: *n*=21.

### Physician Knowledge of Cannabinoids and Therapeutic Considerations

When asked about their current knowledge of cannabinoid molecules (phyto-, syntheto-, and endocannabinoids), 43% of respondents were not confident, while 39% felt “somewhat confident,” and 17% felt “confident.”

Regarding their knowledge of cannabinoid molecules, 74% felt “not confident,” while only 22% felt “somewhat confident” (Table 2).

**Table 2:** Assessing Confidence (23 Respondents).

Survey Question	Response, *n* (%)			
	Very Confident	Confident	Somewhat Confident	Not Confident
“Do you feel confident regarding your current knowledge of the endocannabinoid system in health and disease?”	0	4 (17)	9 (39)	10 (43)
“Do you feel confident regarding your current knowledge of the cannabinoid molecules (phyto-, syntheto-, and endocannabinoids)?”	0	1 (4)	5 (22)	17 (74)
“How confident are you to write a prescription for medical cannabis indicating dosage, frequency of use, and method of administration?”	1 (4)	1 (4)	3 (13)	18 (78)

Among the respondents, 17% believed that there is currently no therapeutic role for use of any cannabinoid in the management of rheumatic diseases, whereas 48% believed there was a role for both pharmacologic cannabinoids and medical cannabis, 17% believed there was a role only for pharmacologic cannabinoids, and 9% only for medical cannabis. Thus, 74% of responders held the opinion that there was some role for cannabinoids in the management of rheumatic disease (Table 3).

**Table 3:** Attitudes Regarding Role for Cannabis in the Treatment of Rheumatic Disorders.

Survey Question	Possible Responses	*n* (%) *
“Is there a role for use of any of the following options for rheumatic conditions?”	Pharma only	4 (17)
	Medical cannabis	2 (9)
	No role	4 (17)
	Both	11 (48)
	Did not answer	2 (9)

“Have you previously recommended a trial of the following?”	Pharma only	0 (0)
	Medical cannabis	11 (48)
	Both	3 (13)
	Never	9 (39)

“Would you recommend a trial of the following?”	Pharma only	4 (17)
	Medical cannabis	2 (9)
	Both	9 (39)
	None	7 (30)
	Did not answer	1 (4)

“Would you write a medical cannabis prescription for a patient failing conventional treatments?”	Yes	19 (83)
	No	3 (13)
	Did not answer	1 (4)

“Would you write a medical cannabis prescription based on patient request regardless of previous treatments?”	Yes	2 (9)
	No	20 (87)
	Did not answer	1 (4)

* Total respondents: *n*=23.

A total of 39% of respondents had never previously recommended any form of cannabinoid treatment to patients, while 48% had previously prescribed medical cannabis, and 13% had previously prescribed either medical cannabis or a pharmacological cannabis preparation (Table 3).

### Specific Responses Regarding Medical Cannabis

Regarding the writing of prescriptions, 78% of respondents were not confident in writing a prescription for medical cannabis when required to indicate dosing, frequency, and method of administration.

Only three Israeli rheumatologists (13% of responders) expressed a suggestion for an initial dose of cannabis, when prescribed, with proposed initial doses of 15–20 g per month. The same physicians also had an idea regarding maximal doses, ranging from 25 to 60 g per month. Only two physicians responded regarding the preferred route of administration.

Medical cannabis would be considered by 82.6% of responders in cases in which conventional modes of treatment had failed, while only 8.7% stated that they would consider prescribing cannabis to a patient requesting such treatment, regardless of previous treatments.

### Narrative Responses

In the context of the current survey, rheumatologists were also asked to respond narratively to some questions, with a sample of responses presented.

In replying to the question regarding “What variables need to be taken into consideration in patients requesting medical marihuana (herbal cannabis) for the treatment of a rheumatic condition?” (question number 11), responses included the following: psychiatric co-morbidities; concern about the drug “leaking” to others; concerns about secondary gain issues; use for non-medical (recreational) reasons; and history of drug or alcohol addiction. Some physicians expressed concern about patients avoiding effective anti-inflammatory treatment and instead using cannabis to avoid pain. Others expressed concerns about driving and motor vehicle accidents. Concerns were also raised about the possible interaction between cannabis and other prescription medications, including opioids.

## DISCUSSION

The results of the current survey indicate that Israeli rheumatologists lack confidence and have significant concerns regarding the use of medical cannabis for patients suffering from rheumatic disorders. This outcome is not surprising and is in line with previously reported data.^12^ It is notable, however, that despite this lack of confidence, those rheumatologists who responded showed relatively high levels of openness to the idea of using medical cannabis. The results indicate that the vast majority of responders (and presumably of rheumatologists in general) lack knowledge regarding basic topics such as dosage and route of administration, and hold concerns related to a broad spectrum of patient-related issues, ranging from psychiatric effects to motor vehicle accidents, as well as concerns pertaining to the societal effects of utilizing cannabis such as “leakage” from patients to others and recreational use. In view of such extensive concerns, it may appear to be surprising that responders nonetheless presented some degree of openness to the possibility of using cannabis. This finding may indicate both the high awareness of the need to improve pain management, and a degree of curiosity regarding a novel field of treatment and research.

As noted above, an identical survey has recently been published regarding the attitudes of Canadian rheumatologists to the use of medical cannabis.^11^ Thus it was possible to compare the current Israeli results with the Canadian ones. While three-quarters of responders in both countries were not confident about their knowledge of cannabinoid molecules or ability to write a prescription for herbal cannabis, this comparison yielded some significant differences between the two countries.

The proportion responding with “no role” for any cannabinoid treatments in Canada and Israel was 45% versus 17%, respectively. The proportion reporting never previously having recommended trial of any cannabinoid was 70% versus 39%, respectively; whereas 30% versus 48%, respectively, believed there was some role for pharmacologic or herbal preparations; and 28% versus 83%, respectively, were “willing to prescribe herbal cannabis if other treatments failed.” The majority of respondents were not confident to write a prescription for herbal cannabis, 90% versus 78%, respectively. The reason why Israeli rheumatologists may be more open to this option is difficult to explain. One possibility may relate to the rapidly changing scope of medical cannabis in Canada, accompanied by rapidly changing legislation, which may increase feelings of uncertainty and concern among Canadian physicians (and rheumatologist in particular). In the past decade the courts in Canada have ruled that a blanket prohibition to use herbal cannabis (marijuana) for medical reasons was unconstitutional, obligating Health Canada to develop regulations to allow possession of herbal cannabis for selected medical reasons. Thus, access to medical herbal cannabis in Canada has been driven by public appeal to the courts, and the medical community has had limited voice in the debate. Additionally, the knowledge that herbal cannabis in Canada is used mostly for musculoskeletal complaints, rather than the anticipated use for end-of-life care, or for management of symptoms related to human immunodeficiency disease, has come as somewhat of a surprise for the health care community. Finally, in the absence of evidence for use of herbal cannabis in the rheumatic diseases, this conservative stance by Canadian rheumatologists can be justified. Other cultural and societal factors may also play a role.^13^

While the current results may not be surprising for many rheumatologists who are well acquainted with the caution and reticence expressed by their colleagues, it is noteworthy that these results describe a snapshot of a situation in flux. As the number of patients being treated with medical cannabis continues to rise, it becomes increasingly likely that a rheumatologist treating patients suffering from musculoskeletal pain will be encountering increasing numbers of patients who either are interested in cannabis treatment or indeed are already being treated with medical cannabis by a specialist from another discipline (e.g. a pain specialist, neurologist, gastroenterologist, etc.).^14^ This reality makes it imperative for the rheumatologist to at least be as well-informed as possible about the effects and outcomes of such treatment, as well as gauging how such treatment may be interacting with other medications the rheumatologist may wish to employ (e.g. narcotics, anticonvulsants, etc.). Moreover, the rheumatologist will have to be aware of the ways in which cannabis may be affecting disease activity measures. Needless to say, similar concerns may also arise in the case of the large group of patients who are using cannabis illicitly,^15^,^16^ either recreationally or as self-medication. In this context, however, things become even more complex due to the non-standard dose of active ingredients used, as well as the co-administration of additional recreational drugs or alcohol. Beyond these practical issues, the legitimate role of cannabis and cannabinoids in the management of rheumatic disorders is an issue well worthy of the attention of the rheumatological community, on both a clinical and a basic scientific level.

As noted, chronic pain and in particular centralized pain, such as that typical of fibromyalgia, continue to be unmet medical needs,^17^ responding in a very incomplete way to current FDA-approved medications. The proportion of patients who achieve worthwhile pain relief (defined as at least 50% pain intensity reduction) is small, typically 10% to 25% more than with placebo, with numbers needed to treat to benefit (NNTs) usually between 4 and 10.^18^,^19^ This challenging problem is not one that should be ignored or relegated to others by rheumatologists. Thus, referring a rheumatological patient to be treated for pain by a specialist in another field (e.g. a pain specialist) is problematic and costly, and may lead to an inconsistent clinical trajectory. In view of these considerations, it seems particularly important that rheumatologist should be as well-acquainted as possible with all aspects of treating pain in rheumatological patients, including whatever knowledge exists regarding the use of cannabinoids.

While the precise mechanisms through which cannabinoids act in chronic pain are far from being well understood, the field of studying the endocannabinoid system has burgeoned into a massive and fascinating area of research.^20^,^21^ While fibromyalgia for instance has long been hypothesized to represent a condition of “endocannabinoid deficiency,”^22^ this view may be an over-simplification, and it appears likely that the end-result of cannabinoid activation represents an intricate interplay between various agonists,^23^ receptors, and breakdown enzymes,^24^ with far-reaching effects regarding pain, sleep, mood, cognition, etc. Current research has not yet delineated with any precision how and when it is best to activate this system for optimal clinical response and what long-term effects this activation will carry with it.^25^,^26^ At a strictly clinical level, using cannabinoids is currently somewhat analogous to “using antibiotics”: one knows they may do a lot of good, but one would really like to know which specific active molecule is being used, at which dose, and for which specific indication.

### Strengths and Limitations of the Current Study

The current study is limited by the relatively low number of responders, although this proportion is similar to previous reports. It is hard to predict whether the sample of rheumatologist who chose to answer the survey is representative of the entire group of Israeli rheumatologists; while it might appear likely that responders may feel more strongly on the topic than non-responders, this might be equally true both for proponents and objectors to the introduction of medical cannabis. The absolute number of participants in the survey also reflects the limited size of the Israeli rheumatological community (total number of rheumatologists 119, at the time of the survey). The strength of this study lies in the implementation of a previously utilized questionnaire, thus enabling a cross-cultural comparison, and in the non-judgmental nature of the questionnaire, making it equally acceptable for rheumatologists in favor of or against the use of cannabis. Notably, the accompanying text, which was addressed to the rheumatologists, was designed to avoid any positive or negative stance toward the issue at hand.

Obviously, current evidence is far from answering these questions in the case of cannabis, and this creates the gap between the increasing need for guidance felt by physicians and the lack of answers. Implementation of further research, including academically sponsored studies (in order to overcome the shortage of industry-sponsored research in this field), is urgently called for in order to narrow the gap.
